# FACIAL-TRUSTWORTHINESS AND BEHAVIORAL-EVALUATION FACILITATE DECISION MAKING AMONG YOUNG AND OLDER ADULTS

**DOI:** 10.1093/geroni/igad104.3340

**Published:** 2023-12-21

**Authors:** Shuyao Liao

**Affiliations:** Peking University, Beijing, Beijing, China (People’s Republic)

## Abstract

Objectives. Older adults prefer non-diagnostic cues (e.g., facial-appearance) than diagnostic behaviors when making decision in trust games. However, previous research on decision-making heavily focused on memory, requiring older adults to remember multiple faces and corresponding behaviors. Due to cognitive deficits and the rarity of repetitive interactions with partners in real-life, older adults may experience limitations in their memory-based decision making. This study employs multi-trial one-shot trust game to investigate the reliance of younger and older adults on non-diagnostic (facial-trustworthiness) and diagnostic (behavioral-evaluation) cues, and whether older adults would learn the rule of basing decisions on diagnostic cue, when both cues are available. Method. 104 younger adults (Mage = 21.38, SD = 2.41) and 105 older adults (Mage = 65.5, SD = 4.15) completed 96-trial one-shot trust game, in which partner’s facial-trustworthiness and behavioral-evaluation were presented simultaneously. Regardless of facial-trustworthiness, decision outcomes were based on behavioral-evaluation. Results. A reinforcement learning model was established, including learning rate and different weights for facial-trustworthiness and behavioral-evaluation. Results reveled that older adults could indeed show rule-based learning, by exhibiting a non-zero learning rate, and an increase in their weights for behavioral-evaluation after learning, although they initially had higher weights for facial-trustworthiness and lower weights for behavioral-evaluation. Discussion. The present study indicated that older adults tended to rely more on facial-appearance initially. However, they could learn the right rule to adjust decision-making resembling those of younger adults. Keywords: decision-making, facial-trustworthiness, behavioral-evaluation, rule-based learning

